# Autologous bone marrow mesenchymal stem cell mitochondrial transplantation in recurrent assisted reproductive technology failure: a randomized controlled trial

**DOI:** 10.1186/s13287-026-05059-5

**Published:** 2026-05-13

**Authors:** Xiaoping Liu, Dandan Wang, Lei Jia, Weixi Chen, Rui Huang, Cong Fang, Cijie Du, Liang Yang, Xingguo Liu, Xiaoyan Liang

**Affiliations:** 1 Reproductive Medicine Research Center, The Sixth Affiliated Hospital, Sun Yat-sen University, Guangzhou, China; 2 GuangDong Engineering Technology Research Center of Fertility Preservation, Guangzhou, China; 3 Biomedical Innovation Center, The Sixth Affiliated Hospital, Sun Yat-sen University, Guangzhou, China; 4 Institute of Development and Regeneration, Guangdong Provincial Key Laboratory of Stem Cell and Regenerative Medicine, Guangdong-Hong Kong Joint Laboratory for Stem Cell and Regenerative Medicine, GIBH-CUHK Joint Research Laboratory on Stem Cell and Regenerative Medicine, GIBH-HKU Guangdong-Hong Kong Stem Cell and Regenerative Medicine Research Centre, China-New Zealand Joint Laboratory on Biomedicine and Health, Guangzhou Institutes of Biomedicine and Health, Chinese Academy of Sciences, Guangzhou, China

**Keywords:** Autologous bone marrow mesenchymal stem cells, Embryo quality, Mitochondrial transplantation, Recurrent ART failure

## Abstract

**Background:**

Mitochondrial dysfunction contributes to poor embryo quality and recurrent assisted reproductive technology (ART) failure. Mitochondrial transplantation (MIT), which involves supplementing oocytes with exogenous mitochondria, has been proposed as a novel strategy to improve ART outcomes. However, both its clinical efficacy and safety remain unclear.

**Methods:**

In this single-center trial, 151 patients with a history of ≥ 2 failed ART cycles provided 1178 metaphase II (MII) oocytes. Sibling oocytes were randomized 1:1 to receive autologous bone marrow mesenchymal stem cells (BMSCs) mitochondria co-injection during intracytoplasmic sperm injection (ICSI) or standard ICSI. The primary outcome was the rate of day-3 good-quality embryos.

**Results:**

MIT significantly accelerated early embryonic cleavage at the 3-cell stage and 5-cell stage, but this morphokinetic alteration did not translate into improvements in good-quality embryo rate, clinical pregnancy rate, or live birth rate. Long-term follow-up of 23 live births revealed no adverse effects, with all offspring exhibiting normal growth and development. Exploratory analysis revealed that oocytes yielding ≥ 70% transferable embryos after MIT harbored an elevated higher burden of medium frequency (0.05–0.5) mtDNA point mutations.

**Conclusions:**

While autologous BMSCs-MIT transiently alters early cleavage kinetics, it does not demonstrate a clinical advantage in unselected patients with recurrent ART failure. Nevertheless, its observed safety profile and the identification of mtDNA mutation burden as a potential predictive biomarker provide a foundation for shifting future MIT research from a universal approach toward precision application in molecularly stratified populations.

*Trial registration* ClinicalTrials.gov registration: NCT03639506

**Supplementary Information:**

The online version contains supplementary material available at10.1186/s13287-026-05059-5.

## Background

Embryo competence is critically dependent on mitochondrial function within the oocyte [1, 2]. Mitochondrial DNA (mtDNA) copy number, structural integrity, and respiratory efficiency govern ATP production, consequently exerting profound regulation on early embryonic development [3]. The growing trend toward delayed childbearing has exacerbated mitochondrial dysfunction related infertility, contributing to the persistent challenge of poor quality embryos and recurrent failure of assisted reproductive technology (ART) among affected patients [4]. Therapeutic options for this population remain limited.

Mitochondrial transplantation (MIT) has emerged as a putative rescue strategy, whereby healthy organelles are microinjected into metaphase II oocytes to restore bioenergetic homeostasis [5, 6]. Yet clinical studies using autologous granulosa‑cell or oogonial‑stem‑cell mitochondria have consistently yielded suboptimal outcomes, primarily due to the synchronous aging of donor mitochondria with host oocytes, compounded by unresolved questions regarding stem cell lineage specification and functional potency [7–9]. Attempts with allogeneic mesenchymal‑stem‑cell mitochondria improve oocyte function in animals but raise immunologic and regulatory concerns in humans [10–12].

Autologous bone marrow mesenchymal stem cells (BMSCs) offer a theoretically optimal mitochondrial source. These cells exhibit negligible immunogenicity and are readily harvested under local anesthesia [13]. Whether BMSC-derived mitochondria can effectively ameliorate oocyte energetic deficits, and which patient subpopulations would derive the greatest clinical benefit from this intervention, have not been established.

We therefore performed the sibling oocyte, randomized controlled trial of autologous BMSC mitochondrial transfer during intracytoplasmic sperm injection (ICSI) in women with a history of ≥ 2 failed ART cycles attributable to persistently poor embryo quality. The trial was designed to (i) evaluate clinical efficacy and safety, (ii) interrogate mtDNA mutation burden as a mechanistic biomarker of treatment response, and (iii) define practical benchmarks for future precision application of MIT.

## Methods

### Ethical approval

This study was approved by the Ethics Committee of the Sixth Affiliated Hospital of Sun Yat-sen University (2016ZSLYEC-001S) and registered at ClinicalTrials.gov (NCT03639506).

### Study design and patient population

Our study exclusively examined females because the disease is only relevant in females. This single-center, randomized, controlled trial was conducted at the Reproductive Medicine Center of The Sixth Affiliated Hospital of Sun Yat-sen University from October 2018 to September 2024. Participants who voluntarily provided written informed consent were enrolled according to the following criteria: (1) Females aged 21–42 years; (2) Anti-Müllerian hormone (AMH) level ≥ 1.1 ng/mL; (3) History of ≥ 2 failed ART cycles; (4) Body mass index (BMI) 18–25 kg/m². Exclusion criteria comprised: (1) Uterine anomalies (including congenital malformations, intrauterine adhesions, or endometrial thickness < 7 mm in previous cycles); (2) Medical contraindications to pregnancy; (3) Surgically confirmed adenomyosis/endometriosis or ultrasound-detected ovarian endometrioma ≥ 2 cm; (4) Untreated hydrosalpinx; (5) Male factors adversely affecting embryo quality (persistent sperm DNA fragmentation > 30% or non-obstructive azoospermia); (6) Use of donor oocyte in ICSI cycles.

### End points

The primary outcome of the study was to compare the rates of day-3 good-quality embryos from the MIT group and the control group. Secondary outcomes included clinical pregnancy rate, live birth rate, and obstetric and neonatal outcomes.

### Randomization

Randomization was performed at the level of the oocyte. Sibling MII oocytes from each patient were randomly assigned in a 1:1 ratio to either the mitochondrial transfer group or the control group using a computer-generated randomization list. Embryo transfers were performed with embryos derived exclusively from one group per transfer. As such, while outcomes related to embryonic development reflect a randomized comparison, clinical pregnancy and live birth rates should be interpreted as non-randomized comparisons given the potential for selection bias at the patient level.

### Ovarian stimulation

All patients underwent controlled ovarian stimulation (COS) using a GnRH agonist long protocol. GnRH agonist (Triptorelin, Ferring, Germany) was administered in the luteal phase of the preceding menstrual cycle. On day 2–3 of menstruation, after confirming successful pituitary down-regulation (E_2_ <50 pg/mL, LH ≤ 5 U/L, endometrial thickness ≤ 5 mm), patients were administered 150–300 IU of recombinant FSH (Gonal-f, Merck-Serono, Switzerland) daily until the trigger day. HCG (Ovidrel, Merck Serono S.p.A., Italy) 0.25 mg was administered for final maturation when at least one leading follicle reached 18 mm or 3 follicles reached 17 mm. Oocyte retrieval was performed transvaginally under ultrasound guidance 36–37 h after hCG injection.

### ICSI procedures

Approximately 4 h after oocyte retrieval, sibling MII oocytes obtained from each patient were randomly allocated to either the mitochondrial transfer (MIT) group or control group at a 1:1 ratio, ensuring that at least one MII oocyte from each patient received MIT intervention. BMSCs were thawed by laboratory technicians on the morning of oocyte retrieval, followed by immediate mitochondrial isolation and purification using the method described by Morimoto et al. [14]. The purified mitochondria were prepared as a mitochondrial suspension. For the MIT group, ICSI was performed by co-injecting approximately 2 pL of mitochondrial suspension with spermatozoa into the oocytes. A representative image of the mitochondrial co-injection procedure is shown in Fig. 2A. Control group oocytes underwent conventional ICSI. All mitochondrial injections were completed within 2 h after isolation to ensure mitochondrial viability. Following microinjection, oocytes were cultured in a Geri^®^ time-lapse incubation (Genea Biomedx, Sydney, Australia) with continuous monitoring until blastocyst stage development.

### Embryo transfer and pregnancy outcomes

A maximum of two day-3 embryos were transferred under ultrasound guidance. Luteal phase support consisted of 400 mg/day micronized progesterone (Utrogestan, Besins Healthcare, France) and 20 mg/day dydrogesterone (Duphaston, Abbott, Netherlands) administered for 17 days post-oocyte retrieval. Fresh embryo transfers were cancelled if premature progesterone elevation (> 1.5 ng/mL on hCG trigger day) or endometrium < 8 mm, with subsequent embryo vitrification.

Endometrial preparation involved hormone replacement therapy initiated on cycle day 2–3 with oral estradiol valerate (Progynova, Delpharm Lille, France) 4 mg/day. Upon achieving endometrial thickness ≥ 8 mm, dydrogesterone 30 mg/day (Duphaston) and micronized progesterone 600 mg/day (Utrogestan) were administered. Cleavage-stage (day 3) or blastocyst (day 5) embryos were transferred 3 or 5 days later, respectively, with ≤ 2 cleavage-stage embryos or 1 blastocyst per transfer.

Serum β-hCG levels were quantified 12–14 days post-transfer to confirm biochemical pregnancy. Clinical pregnancy required ultrasound visualization of intrauterine gestational sac with fetal cardiac activity. Live birth denoted delivery of ≥ 1 viable neonate (≥ 28 weeks’ gestation). Follow-up of the newborn was conducted with all patients for 6 months.

### Sample size calculation and statistical analysis

The sample size was determined to identify a 15% difference in the rate of good-quality embryos, from 30% in the Control group to 45% in the MIT group. The anticipated 15% absolute improvement in good-quality embryo rate was primarily determined based on preliminary data from our center, which indicated that this effect size represented a clinically meaningful threshold in patients with recurrent ART failure. This expected effect size was also consistent with the magnitude of improvement reported in early clinical study of mitochondrial transfer in similar poor-prognosis populations [15]. This calculation was based on a two-tailed test with 80% power (beta error 0.2) and a 95% confidence interval (alpha error 0.05). Using the formula for paired samples, the required sample size was calculated to be approximately 160 pairs. Considering potential sample loss or data missing in the actual research, it is recommended to increase the sample size by 10%, resulting in a suggested 176 pairs for the study.

Continuous variables were presented as mean ± SD or median and interquartile value (IQR), based on normal or abnormal sample distribution, respectively. For the intrasubject comparison between both groups, a paired-samples t test or Wilcoxon Signed Ranks Test was done. The comparison of rates between the two groups was performed using either the chi-square test or Fisher’s exact test, depending on the appropriateness of the data assumptions. A P value of < 0.05 was considered to be statistically significant.

### BMSCs procurement and culture

BMSCs were obtained from female patients with written informed consent and no immunological diseases. Patients underwent bone marrow puncture to obtain bone marrow under local infiltration anesthesia. BMSCs were obtained by density-gradient centrifugation isolation and cultured in Dulbecco’s modified Eagle’s medium (DMEM, Gibco) containing 10% fetal bovine serum (FBS, Gibco), 1% penicillin/streptomycin (Gibco), and 10 ng/ml recombinant basic fibroblast growth factor (PEPROTECH). BMSCs were passaged upon reaching approximately 80–90% confluence.

### Assessment of isolated mitochondria

BMSCs were used to isolate mitochondria. The viability of isolated mitochondria was determined by fluorescence microscopy and flow cytometry. Isolated mitochondria were suspended in TMRM (Invitrogen) buffer solution and incubated with medium, TMRM, and TMRM+carbonyl cyanide 3-chlorophenylhydrazone (CCCP), respectively, at 37 °C for 20 min. CCCP can decrease the potential of the mitochondria membrane by inhibiting oxidative phosphorylation. After being rinsed twice, the mitochondria pellets were resuspended and then analyzed by Cytoflex flow cytometry (Beckman Coulter). The mitochondria were stained with 10000X Mito-Tracker deep red, rinsed twice, and imaged using a confocal microscope (Leica TCS SP8). Transmission electron microscopy (TEM) was used to examine the mitochondrial ultrastructure. The mitochondria pellets were fixed with 2.5% glutaraldehyde in 0.1 M PBS at 4 °C for 2 h and then with 1% OsO4 for 1 h. The samples were washed, dehydrated using graded acetone, and embedded in EPON™–Araldite resin. After polymerization, 60 nm sections were cut with an ultramicrotome, stained with uranyl acetate and lead citrate, and observed using a JEM-1400 transmission electron microscope at 100 kV.

### Analysis of mtDNA mutations

Following obtainment of patient informed consent, BMSCs and discarded immature oocytes from 6 patients were collected during ICSI procedures. The number of immature oocytes collected per female patient ranged from 1 to 4. Each individual immature oocyte was processed as a separate sample. MtDNA mutation analysis for human oocytes was performed according to the detailed methodology described by Yang et al. [16]. Briefly, oocyte samples were lysed in 20 µL of lysis buffer at 56 °C for 45 min, followed by incubation at 95 °C for 10 min. Four pairs of primers were used to amplify regions covering the entire mtDNA sequence (region 3561–9794, region 9795–14,567, region 14,562–139, and region 115–3560). The amplified sequences for each sample were pooled and sent for sequencing. The resulting ddRAD (double-digest restriction-associated DNA) libraries were submitted to Berry Genomics Co., Ltd. Sequencing was performed on the Illumina Hiseq4000 platform using 150-bp paired-end reads, generating approximately 3 Gb of data per sample. For analyzing mtDNA mutation of human BMSCs, the total genome was first extracted using a TIANamp Genomic DNA kit (DP304, TIANGEN, Beijing, China). Then the mtDNA was amplified and sequencing using the same methodology previously employed for oocyte assessment. Analysis was conducted as described by Yang et al. [16].

## Results

### Patients’ characteristics

**Fig. 1:**
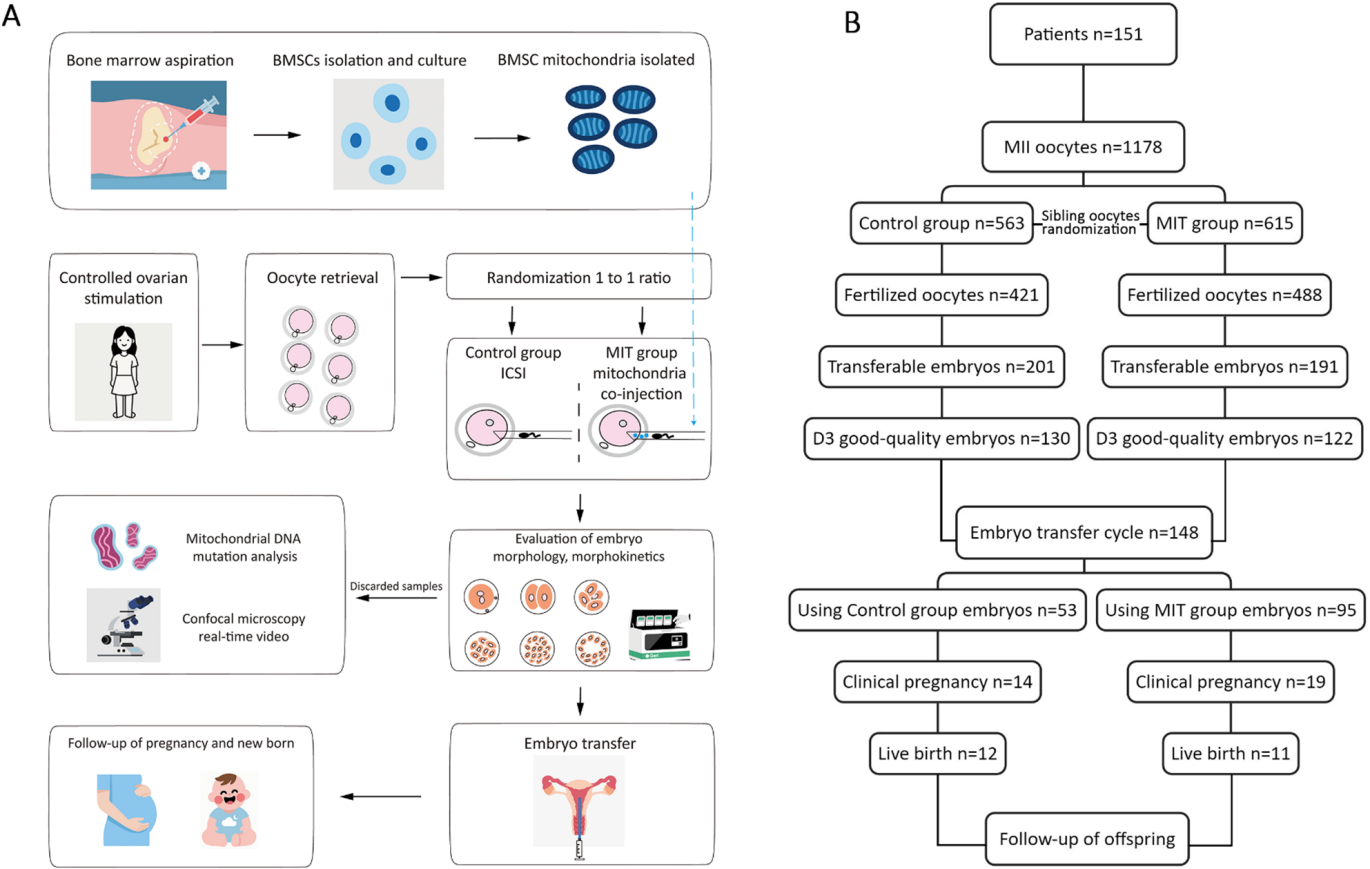
**A** Schematic drawing representing the complete procedure. This figure was created by the authors using Adobe Illustrator. Partial graphical elements were generated by Doubao AI (ByteDance Inc.) and further modified by the authors. **B** Study flow diagram

Figure 1 showed the complete procedure of this study. A total of 151 patients were enrolled in this study. The participants had a mean number of 2.55 ± 1.01 previous failed ART cycles, with baseline characteristics showing a mean age of 35.12 ± 5.33 years, body mass index of 21.85 ± 2.45 kg/m², and infertility duration of 5.84 ± 2.98 years. The mean serum anti-Müllerian hormone (AMH) level was 3.27 ± 2.45 ng/mL, while the mean antral follicle count (AFC) was 13.22 ± 7.89. All enrolled patients underwent ovarian stimulation using a GnRH agonist protocol. The total gonadotropin (Gn) dosage administered averaged 2349.67 ± 975.68 IU over a mean duration of 10 ± 2.62 treatment days. The mean number of oocytes retrieved per patient was 12.1 ± 7.46 (Table 1).

**Table 1:** Characteristics of study population (*n* = 151)

	Mean ± SD
Previous ART failed cycles	2.55 ± 1.01
Age (years)	35.12 ± 5.33
Duration of infertility (years)	5.84 ± 2.98
BMI (kg/m^2^)	21.85 ± 2.45
Basal FSH (IU/L)	6.93 ± 2.16
Basal E_2_ (pg/mL)	39.4 ± 17.53
Basal LH (U/L)	4.34 ± 1.58
AMH (ng/mL)	3.27 ± 2.45
AFC	13.22 ± 7.89
Total Gn dose (IU)	2349.67 ± 975.68
Duration of Gn stimulation (days)	10 ± 2.62
Serum E_2_ level on trigger day (pg/mL)	2352.16 ± 1969.66
Serum LH level on trigger day (U/L)	1.89 ± 2.4
Number of oocytes retrieved	12.1 ± 7.46

ART: assisted reproductive technology; BMI: body mass index; FSH: follicle stimulating hormone; E_2_: estradiol; LH: luteinizing hormone; AMH: anti-müllerian hormone; AFC: antral follicle count; Gn: gonadotropin

### Embryonic developmental outcomes

**Fig. 2:**
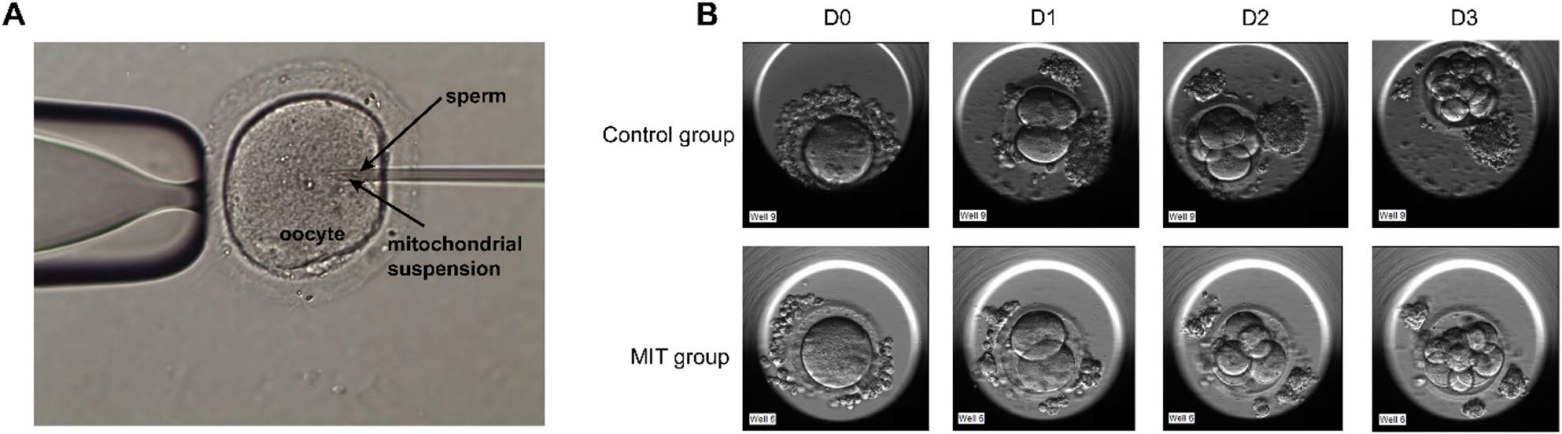
Mitochondrial injection during ICSI and representative embryonic development. **A** Image of mitochondrial co-injection during intracytoplasmic sperm injection (ICSI). **B** Representative images of embryos derived from Control and MIT oocytes at indicated days after ICSI (D0–D3)

Representative images of embryos derived from Control and MIT oocytes are shown in Fig. 2B. In vitro embryo developmental outcomes are presented in Table 2. A total of 563 MII oocytes were allocated to the Control group and 615 to the MIT group. Comparable 2 pronucleus (PN) fertilization rates were observed between groups. The MIT group demonstrated significantly lower 2PN cleavage rates (75% [50%, 100%]) compared to the Control group (100% [55%, 100%]; *P* = 0.015). No statistically significant differences were observed in the rates of transferable embryo or good-quality embryo between the groups. We compared embryo outcomes between two groups in young and advanced maternal age populations, using 35 years as the cutoff. In the young population, there were no differences in 2PN fertilization rate, cleavage rate, transferable embryo rate, or good-quality embryo rate between the two groups. In the advanced maternal age population, the MIT group exhibited a significantly higher 2PN fertilization rate than the control group, while cleavage rate, transferable embryo rate, and good-quality embryo rate were comparable to the control group (Supplementary Table 1).

**Table 2:** Comparison of lab results between the two groups

	Control group	MIT group	*P* value
Total number of MII oocytes	563	615	-
No. of MII oocytes	5.17 ± 3.13	5.60 ± 2.90	0.002^a*^
No. of 2PN	3.81 ± 2.35	4.28 ± 2.56	0.004^a*^
No. of cleavage 2PN	2.83 ± 1.87	2.94 ± 2.14	0.521^a^
No. of transferable embryos	1.82 ± 1.50	1.70 ± 1.48	0.477^a^
No. of good-quality embryos	1.17 ± 1.28	1.08 ± 1.23	0.497^a^
Rate of 2PN (%)	80 (60, 100)	80 (67, 100)	0.738^b^
Rate of 2PN cleavage (%)	100 (55, 100)	75 (50, 100)	0.015^b*^
Rate of transferable embryos (%)	67 (33, 100)	60 (0, 100)	0.204^b^
Rate of good-quality embryos (%)	33 (0, 67)	33 (0, 60)	0.355^b^

^a^Using Paired t - test; values are presented as mean ± SD

^b^Using Wilcoxon Signed Ranks Test; values are presented as median (interquartile range)

*Results are significantly different between the two groups

### Embryonic morphokinetic analysis

Morphokinetic parameters were analyzed using time-lapse imaging incubator. Oocytes in the MIT group exhibited shorter time intervals than the Control group in reaching each developmental stage prior to the 7-cell (t7) stage, with particularly pronounced acceleration observed at both the 3-cell (t3; 35.81 ± 5.62 h vs. 40.97 ± 4.01 h, *P* = 0.022) and 5-cell (t5; 47.03 ± 9.96 h vs. 57.02 ± 6.71 h, *P* = 0.017) stages. Conversely, the MIT group displayed delayed progression through the 8-cell (t8) and 9-cell (t9) stages, though these differences did not reach statistical significance. Similarly, blastocyst formation timing was prolonged in the MIT group compared to the control group, albeit without statistical significance. More detailed morphokinetic parameters are presented in Table 3.

**Table 3:** Morphokinetic parameters for embryos between the two groups

	Control group	MIT group	*P* value
TPB_2_	5.40 ± 1.32	5.31 ± 0.95	0.874
TPNa	15.11 ± 4.18	12.84 ± 3.77	0.181
TPNf	27.16 ± 2.57	25.54 ± 3.35	0.182
T2	29.76 ± 2.49	27.85 ± 3.63	0.133
T3	40.97 ± 4.01	35.81 ± 5.62	0.022*
T4	42.04 ± 3.97	40.14 ± 3.48	0.257
T5	57.02 ± 6.71	47.03 ± 9.96	0.017*
T6	59.52 ± 8.68	53.21 ± 10.75	0.166
T7	64.15 ± 9.63	60.24 ± 10.51	0.428
T8	65.97 ± 14.37	69.89 ± 19.63	0.678
T9	77.58 ± 16.56	78.85 ± 20.14	0.916
TM	101.98 ± 4.96	99.57 ± 5.12	0.482
TsB	112.40 ± 7.31	117.89 ± 10.59	0.387
TB	120.85 ± 10.75	122.82 ± 8.13	0.749
TEB	132.97 ± 1.79	131.57 ± 9.88	0.821

Values are mean ± SD time in hours. TPB_2_: Time of the second polar body extrusion; TPNa: Time of pronuclear appearance; TPNf: Time of pronuclear fading; T2-T9: Time of blastomere cleavage to the 2-, 3-, 4-, 5-, 6-, 7-, 8-, and 9-cell stages; TM: Time of reaching the morula; TsB: Time of starting blastocyst; TB: Time of blastocyst formation; TEB: Time of reaching expanded blastocyst

*Results are signifcantly diferent between the two groups

### Pregnancy outcomes

A total of 148 patients underwent embryo transfers during the study period, with all embryo transfers strictly using embryos from a single group (either Control or MIT). A total of 53 patients underwent embryo transfer with Control group embryos, including 25 fresh embryos and 28 frozen-thawed embryos. A total of 95 patients underwent embryo transfer with MIT group embryos, including 59 fresh embryos and 36 frozen-thawed embryos. Both groups exhibited comparable biochemical pregnancy rates (BPR) and clinical pregnancy rates (CPR). However, the live birth rate (LBR) differed between groups, with 22.64% (Control group) versus 11.58% (MIT group). Further analysis revealed similar rates in BPR, CPR, or LBR between MIT and Control groups undergoing fresh embryo transfers. In contrast, during frozen-thawed embryo transfers, the Control group showed higher BPR, CPR, and LBR. More detail outcomes were presented in Table 4.

**Table 4:** Pregnancy outcomes after transferring embryos from Control and MIT groups

	Control group	MIT group	*P* value
Number of fresh embryo transfer cycles	25	59	
Biochemical pregnancy rate (%)	24.00 (6/25)	27.12 (16/59)	0.766
Clinical pregnancy rate (%)	20.00 (5/25)	23.73 (14/59)	0.709
Miscarriage rate (%)	20.00 (1/5)	42.86 (6/14)	0.603^a^
Live birth rate (%)	16.00 (4/25)	13.56 (8/59)	0.744^a^
Number of frozen-thawed embryo transfer cycles	28	36	
Biochemical pregnancy rate (%)	32.14 (9/28)	22.22 (8/36)	0.373
Clinical pregnancy rate (%)	32.14 (9/28)	13.89 (5/36)	0.080
Miscarriage rate (%)	11.11 (1/9)	40.00 (2/5)	0.505^a^
Live birth rate (%)	28.57 (8/28)	8.33 (3/36)	0.047^a*^
Total number of embryo transfer cycles	53	95	
Biochemical pregnancy rate (%)	28.30 (15/53)	25.26 (24/95)	0.687
Clinical pregnancy rate (%)	26.42 (14/53)	20.00 (19/95)	0.369
Miscarriage rate (%)	14.29 (2/14)	42.11 (8/19)	0.131^a^
Live birth rate (%)	22.64 (12/53)	11.58 (11/95)	0.075

Statistical methods employed chi-square test, unless otherwise specified

^a^Using Fisher’s exact test

*Results are significantly different between the two groups

### Obstetric and neonatal outcomes

Among 23 clinical pregnancies achieved during the study period, 12 patients underwent embryo transfer with the Control group embryos and 11 patients underwent embryo transfer with the MIT group embryos. In the pregnant women with control embryo transfer, twin pregnancies occurred in 3 cases (25.00%), with 1 preterm birth (8.33%) and 10 cesarean deliveries (83.33%). Neonates derived from control embryos had a mean gestational age of 267.33 ± 7.18 days, birth height of 48.67 ± 2.32 cm, and birth weight of 2886.67 ± 418.43 g, with low birth weight (< 2500 g) observed in 3 neonates (20.00% of live births). And, pregnant women with MIT embryo transfer exhibited 1 twin pregnancy (9.09%), 4 preterm births (36.36%), and 7 cesarean deliveries (63.64%). Neonates derived from MIT embryos exhibited a mean gestational age of 268.73 ± 11.31 days, with birth parameters measuring 48.88 ± 4.67 cm in height and 3058.33 ± 723.60 g in weight. Low birth weight (< 2500 g) was observed in 4 neonates (33.33%). Notably, no congenital malformations were reported in either group (Table 5). We conducted a long-term follow-up study on the 23 live births. As of May 2025, none of the children had experienced iatrogenic death, no serious illnesses had occurred, and all the children were performing well academically and maintaining good health in their daily lives (Supplementary Tables 2&3).

**Table 5:** Obstetric and neonatal outcomes from Control and MIT groups

	Control group	MIT group	*P* value
Number of pregnant women	12	11	
Twin pregnancies	3 (25.00%)	1 (9.09%)	0.590^a^
Preterm birth	1 (8.33%)	4 (36.36%)	0.342^a^
Cesarean delivery	10 (83.33%)	7 (63.64%)	0.371^a^
Gestational age at delivery (days)	267.33 ± 7.18	268.73 ± 11.31	0.725
Height at birth (cm)	48.67 ± 2.32	48.88 ± 4.67	0.887
Birth weight (g)	2886.67 ± 418.43	3058.33 ± 723.60	0.447
Low birth weight (< 2500 g)	3 (20.00%)	4 (33.33%)	0.667^a^

Statistical methods employed Student’s t test, unless otherwise specified

^a^Using Fisher’s exact test

### BMSC characterization and mitochondrial integrity assessment

**Fig. 3:**
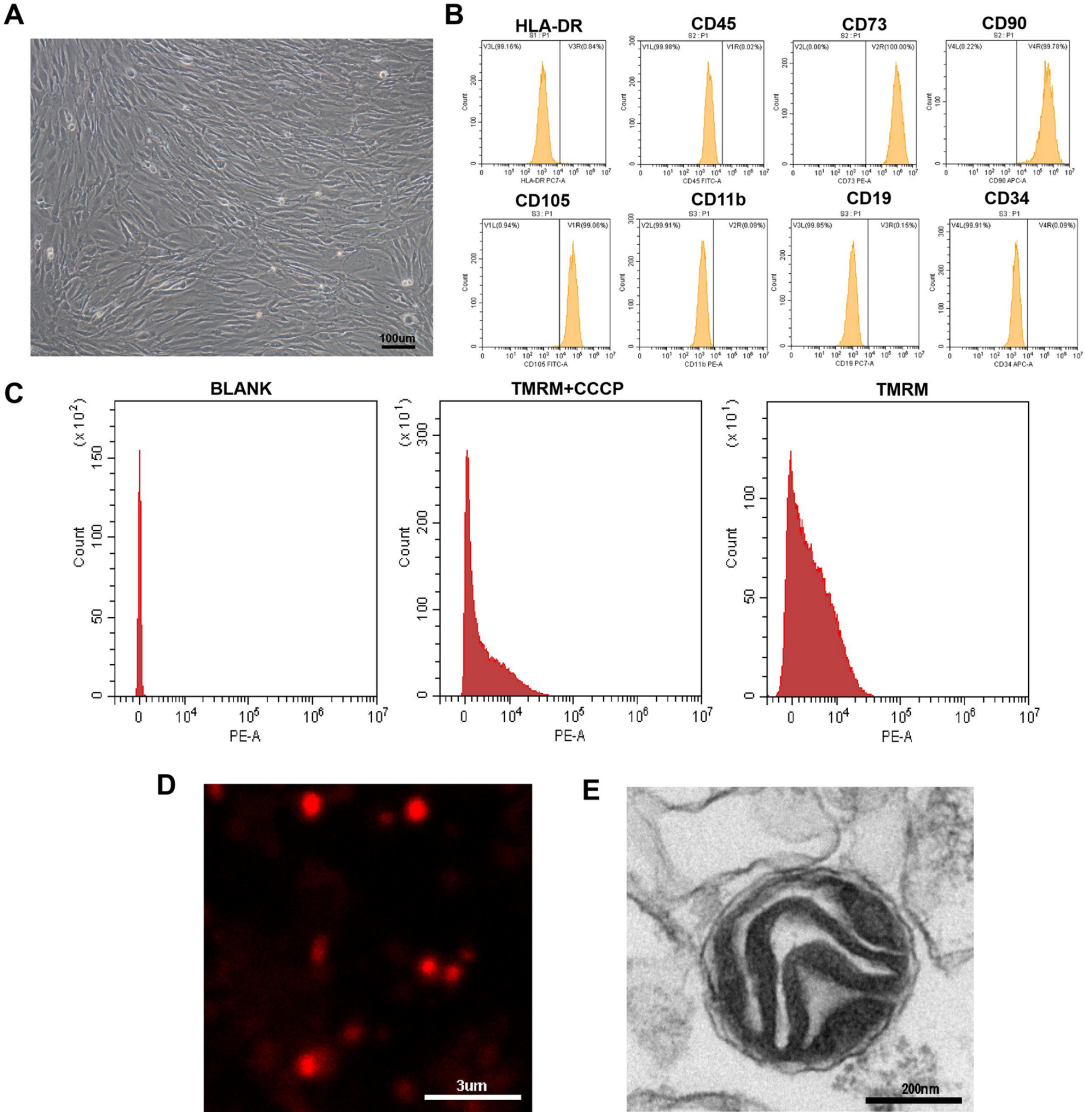
Identification of BMSCs and isolated mitochondria. **A** Light microscope image of BMSCs morphology. Scale bar=100 μm. **B** Flow cytometric identification of BMSCs. **C** Measurement of the membrane potential of mitochondria isolated from BMSCs by flow cytometry. **D** Detection of mitochondrial inner membrane potential by TMRM immunofluorescence. Scale bar=3 μm. **E** Electron microscopy of mitochondria isolated from BMSCs. Scale bar=200 nm

Isolated BMSCs displayed classic fibroblast-like morphology and expressed established immunophenotypic markers of mesenchymal stem cells (positive for CD73, CD90, CD105; negative for HLA-DR, CD45, CD19, CD11b, and CD34) (Fig. 3A, B). Mitochondria isolated from BMSCs exhibited high mitochondrial inner membrane potential as measured by TMRM staining, which was abolished by CCCP uncoupler treatment, confirming functional integrity (Fig. 3C, D). Ultrastructural analysis via electron microscopy revealed intact double membranes with well-preserved cristae architecture (Fig. 3E).

### Analysis of mtDNA point mutations

**Fig. 4:**
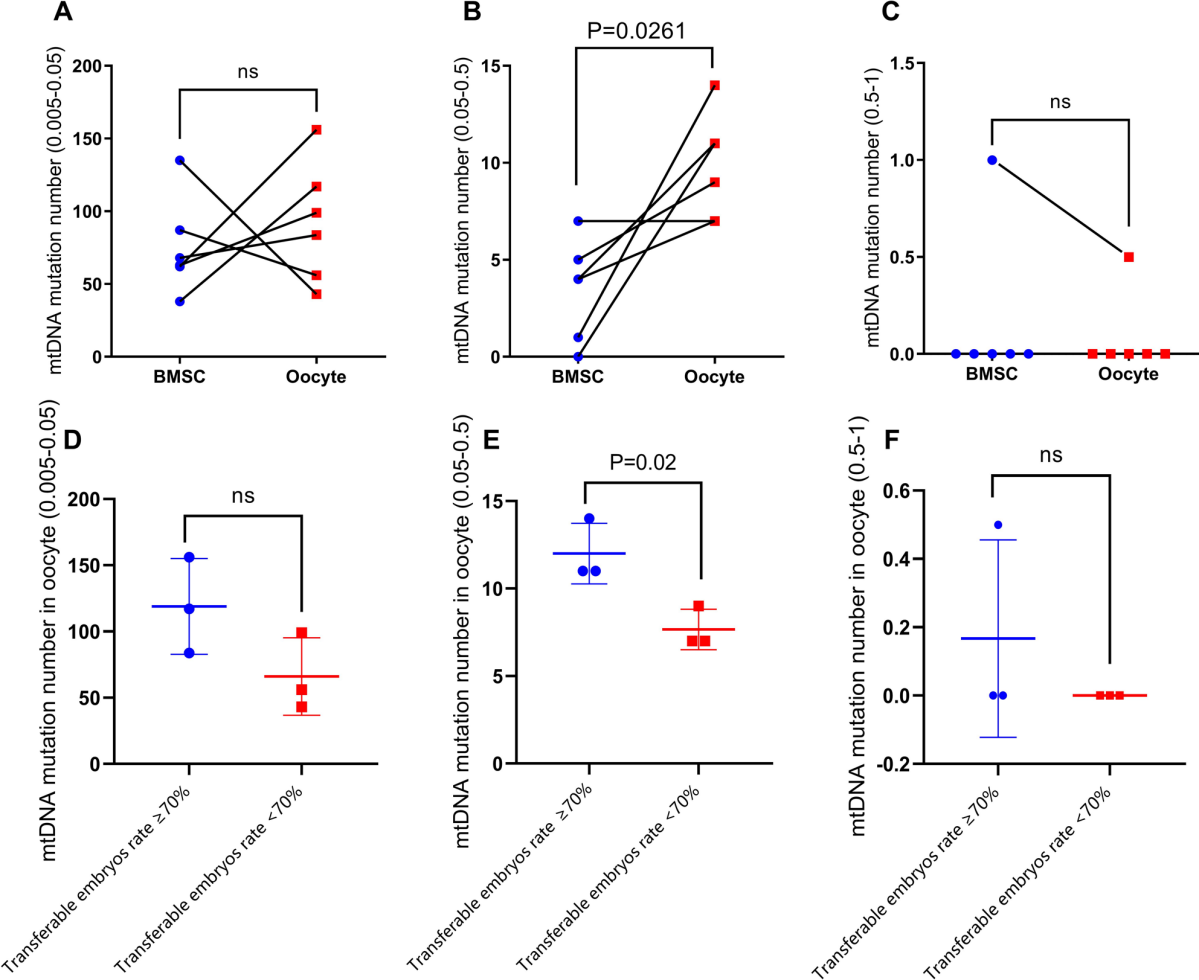
Analysis of mtDNA point mutations. **A** Number of mtDNA point mutations with low frequency with (0.005–0.05) between BMSCs and discarded oocytes (*n* = 6). **B** Number of mtDNA point mutations with medium frequency (0.05–0.5) between BMSCs and discarded oocytes (*n* = 6). **C** Number of mtDNA point mutations with high frequency (0.5-1.0) between BMSCs and discarded oocytes (*n* = 6). **D** Number of mtDNA point mutations with low frequency (0.005–0.05) in discarded oocytes from different MIT-transferable embryos rates group (*n* = 3). **E** Number of mtDNA point mutations with frequency with medium frequency (0.05–0.5) in discarded oocytes from different MIT-transferable embryos rates group (*n* = 3). **F** Number of mtDNA point mutations with high frequency (0.5-1.0) in discarded oocytes from different MIT-transferable embryos rates group (*n* = 3)

To examine cell-type-specific variations in mtDNA of autologous BMSCs and oocytes from patients, we performed comprehensive mtDNA point mutation profiling. To account for potential sequencing artifacts, we established a stringent threshold of > 0.005 mutation frequency for inclusion in our analysis. The identified mtDNA point mutations were systematically classified into three distinct frequency ranges: low-frequency (0.005–0.05), medium-frequency (0.05–0.5), and high-frequency (0.5-1.0) variants. Comparative analysis revealed that autologous oocytes contained a significantly higher burden of medium-frequency mtDNA point mutations (0.05–0.5 range) compared to paired BMSCs (*p* < 0.01, Fig. 4B). Subsequently, the 6 patients were stratified into two groups based on their MIT transferable embryo rate, using a threshold of ≥ 70%. MtDNA point mutation analysis revealed that the group with an MIT-transferable embryo rate ≥ 70% exhibited a significant increase in medium-frequency mtDNA point mutations (0.05–0.5) within oocytes relative to the low-responder group (Fig. 4E).

## Discussion

In this randomized controlled trial, we evaluated the efficacy and safety of intracytoplasmic MIT using autologous BMSCs in patients with recurrent ART failure. Our findings showed that MIT significantly accelerated early-stage embryonic cleavage, specifically at the 3-cell and 5-cell stages. However, recent insights into DNA replication dynamics during early embryogenesis [17] suggest that such acceleration should not be equated with improved developmental competence. In that study, the emergence of a somatic-like replication timing program at the 4-cell stage was accompanied by persistently slow replication forks and increased replication stress, which in turn led to chromosome segregation errors during the subsequent division. Viewed in this context, the accelerated early cleavage observed in our MIT-treated embryos may reflect altered cell cycle progression rather than a genuine enhancement of embryo quality, an interpretation consistent with the absence of improvement in later stage development or clinical outcomes.

Importantly, longitudinal follow-up confirmed the safety of the procedure. All live-born infants derived from MIT-treated embryos exhibited normal growth, cognitive development, and health status, with no adverse obstetric or neonatal outcomes. These findings suggested the clinical feasibility and biological safety of autologous mitochondrial transfer, a critical prerequisite for its continued development as an innovative therapeutic approach in reproductive medicine.

Our results are consistent with Labarta et al. who also observed limited efficacy of MIT in improving embryo quality and reproductive outcomes [9]. However, our study differs from previous work in two key aspects: the use of autologous BMSCs as a distinct mitochondrial source, and the inclusion of comprehensive mtDNA mutational profiling. Despite these methodological refinements, we observed no improvement in embryo quality or reproductive outcomes. These results are in line with the position of the ESHRE Add-ons working group that mitochondrial transfer should not currently be offered as an adjunct in clinical practice [18]. Notably, the exploratory finding that a medium-frequency mtDNA mutation burden may serve as a potential biomarker of response suggests that future investigations should prioritize patient stratification over universal application. Conversely, Morimoto et al. reported more optimistic outcomes using autologous oogonial stem cell-derived mitochondria [14]. The discrepancy may be explained by methodological differences, such as the source of mitochondria and the technical quality of the isolation procedures. Notably, our study used well-characterized BMSCs with verified mitochondrial integrity, thus providing a high-quality mitochondrial source with documented structural and functional viability.

Despite these precautions, we observed delayed developmental kinetics beyond the 8-cell stage in the MIT group, suggesting that transient metabolic support during early cleavage may be insufficient to sustain developmental competence through blastocyst formation. This is further complicated by the biological fact that mitochondrial biogenesis and mtDNA replication are largely suppressed at the MII stage and are only reactivated at the peri-implantation stage [19, 20]. Thus, the timing and functional integration of transplanted mitochondria may limit their utility in supporting later developmental milestones.

Our study also addressed a key mechanistic barrier, that of the intracellular distribution and integration of exogenous mitochondria. Our confocal imaging revealed aggregation of transplanted mitochondria into perinuclear clusters (Supplementary video), suggesting that current microinjection-based delivery systems may not allow efficient dispersion or integration into the recipient mitochondrial network. This lack of homogeneity likely impairs the capacity of exogenous mitochondria to complement endogenous energy production.

Another innovative aspect of our study was the systematic integration of mtDNA mutation profiling. We identified a significantly higher burden of medium frequency mtDNA point mutations in oocytes as compared to autologous BMSCs, potentially accounting for the suboptimal metabolic performance observed in patients with recurrent ART failure [6]. Notably, patients with higher MIT-transferable embryo rates exhibited an even higher burden of such mutations, suggesting a threshold effect: oocytes with moderate mitochondrial dysfunction may derive more benefit from supplementation, whereas oocytes with minimal or severe dysfunction may not exhibit measurable improvements.

This study has several limitations. First, although sibling oocyte randomization minimized inter-patient variability, embryo transfer decisions were based on embryo availability and quality, possibly introducing selection bias. Consequently, our clinical outcome comparisons are observational and should not be interpreted as randomized evidence. Second, subgroup stratification by mitochondrial function or metabolic phenotype was not performed, limiting our ability to identify responsive subpopulations. Nevertheless, descriptive analysis revealed case-level benefit, especially in older patients or those with poor ovarian reserve, which suggested a potential niche application for MIT as a rescue therapy. Additionally, although no congenital malformations or iatrogenic complications were observed, more extensive postnatal surveillance and larger cohorts will be essential to confirm long-term safety and rule out rare or late-onset adverse events. Confounding variables, such as endometrial receptivity, immunological milieu, and fresh versus frozen cycle differences, may have also influenced the clinical outcomes [21–23].

Future MIT research should adopt a multidimensional innovation strategy encompassing donor optimization, delivery system refinement, clinical protocol personalization, and technological integration. First, donor selection should prioritize high-functionality mitochondria or gene-edited variants engineered to meet oocyte-specific metabolic requirements. Concurrently, advanced delivery platforms, such as exosome-based systems leveraging natural intercellular communication mechanisms, could enable precise mitochondrial transfer with enhanced integration efficiency and reduced mechanical disruption. Clinically, single-cell metabolomics should be implemented to stratify patients by age, ovarian reserve, and mitochondrial dysfunction profiles, thereby facilitating personalized transplantation strategies, particularly for mitochondria-deficient subgroups. Technologically, innovations should focus on optimizing nanocarriers and mitochondrial targeting systems, while integrating multi-omics approaches to dynamically monitor mitochondrial genomic stability and functional integration. These synergistic advancements may help establish a comprehensive framework spanning donor screening, precision delivery, real-time monitoring, and outcome evaluation, ultimately driving the clinical translation of MIT as a tailored therapeutic intervention in reproductive medicine.

## Conclusions

In conclusion, while autologous BMSCs-derived MIT represented a biologically rational strategy to address oocyte mitochondrial dysfunction, our trial did not demonstrate improvements in embryo quality or clinical reproductive outcomes for unselected patients with recurrent ART failure. These findings are consistent with the existing body of negative evidence and lend further support to current clinical recommendations against its routine application. However, this study established the long-term biological safety of the procedure and identified medium-frequency mtDNA point mutations as a potential predictive biomarker. These insights may offer a shift in our understanding, moving the field from a universal strategy toward a targeted molecular intervention. Future research should focus on rigorous patient stratification and advanced transfer methods to establish whether MIT can fulfill its promise as a precision therapeutic tool in reproductive medicine.

## Supplementary Information

Below is the link to the electronic supplementary material.

Supplementary Material 1.

Supplementary Material 2.

## Data Availability

The Reproductive Medicine Center, Sixth Affiliated Hospital, Sun Yat-sen University is the data controller for the data in this study. Data access requests should first contact the corresponding author (liangxy2@mail.sysu.edu.cn) to discuss data of interest and to obtain approval. Data for all outcomes in this paper can be requested. The timeline between requesting data and approval of data requests is 3 months. Data will be provided within 3 months of approval. The raw sequence data reported in this paper have been deposited in the Genome Sequence Archive (Genomics, Proteomics & Bioinformatics 2025) in National Genomics Data Center (Nucleic Acids Res 2025), China National Center for Bioinformation / Beijing Institute of Genomics, Chinese Academy of Sciences (GSA-Human: HRA013110) that are publicly accessible at https://ngdc.cncb.ac.cn/gsa-human.

## Abbreviations

mtDNA: Mitochondrial DNA
ART: Assisted reproductive technology
MIT: Mitochondrial transplantation
BMSCs: Bone marrow mesenchymal stem cells
ICSI: Intracytoplasmic sperm injection
COS: Controlled ovarian stimulation
CCCP: Carbonyl cyanide 3-chlorophenylhydrazone
TEM: Transmission electron microscopy
ddRAD: Double-digest restriction-associated DNA
AMH: Anti-Müllerian hormone
AFC: Antral follicle count
Gn: Gonadotropin
PN: Pronucleus
BPR: Biochemical pregnancy rate
CPR: Clinical pregnancy rate
LBR: Live birth rate
