# Supplementary material for: Autologous bone marrow mesenchymal stem cell mitochondrial transplantation in recurrent assisted reproductive technology failure: a randomized controlled trial
Source: Stem Cell Res Ther. 2026 May 13;17:244. doi: 10.1186/s13287-026-05059-5 (PMC13339468; doi:10.1186/s13287-026-05059-5)
Supplement: Supplementary file 2 — Supplementary Material 2. [file 13287_2026_5059_MOESM2_ESM.docx]

Supplementary Table 1 Comparison of lab results between the two groups according to age.

|  |  | Control group | MIT group | *P* value |
| --- | --- | --- | --- | --- |
| Age<35years | No. of 2PN | 3.83 ± 2.15 | 3.98 ± 2.33 | 0.506^a^ |
|  | No. of cleavage 2PN | 2.74 ± 1.67 | 2.59 ± 1.96 | 0.515^a^ |
|  | No. of transferable embryos | 1.84 ± 1.47 | 1.41 ± 1.30 | 0.058^a^ |
|  | No. of good-quality embryos | 1.24 ± 1.32 | 1.03 ± 1.15 | 0.285^a^ |
|  | Rate of 2PN (%) | 89 (60, 100) | 80 (67, 100) | 0.153^b^ |
|  | Rate of 2PN cleavage (%) | 86 (50, 100) | 67 (33, 100) | 0.066^b^ |
|  | Rate of transferable embryos (%) | 67 (29, 100) | 50 (0, 100) | 0.170^b^ |
|  | Rate of good-quality embryos (%) | 33 (0, 67) | 33 (0, 60) | 0.648^b^ |
| Age≥35years | No. of 2PN | 3.78 ± 2.57 | 4.61 ± 2.79 | < 0.001^a*^ |
|  | No. of cleavage 2PN | 2.94 ± 2.09 | 3.35 ± 2.29 | 0.096^a^ |
|  | No. of transferable embryos | 1.78 ± 1.54 | 2.02 ± 1.62 | 0.342^a^ |
|  | No. of good-quality embryos | 1.10 ± 1.24 | 1.14 ± 1.31 | 0.835^a^ |
|  | Rate of 2PN (%) | 75 (52, 100) | 80 (67, 100) | 0.029^b*^ |
|  | Rate of 2PN cleavage (%) | 100 (67, 100) | 79 (62, 100) | 0.102^b^ |
|  | Rate of transferable embryos (%) | 67 (37, 100) | 67 (33, 100) | 0.735^b^ |
|  | Rate of good-quality embryos (%) | 33 (0, 67) | 25 (0, 65) | 0.340^b^ |

^a^ Using Paired t - test; values are presented as mean ± SD.

^b^ Using Wilcoxon Signed Ranks Test; values are presented as median (interquartile range).

* Results are signifcantly diferent between the two groups.

Supplementary Table 2 Detailed embryonic outcomes of patients with live births following MIT embryo transfer

| Patient number | Age (y) | AMH (ng/mL) | No. of oocytes retrieved | MIT group | | | | | Control group | | | | | Child's age (As of 2025) | Intelligence quotient and health condition |
| --- | --- | --- | --- | --- | --- | --- | --- | --- | --- | --- | --- | --- | --- | --- | --- |
|  |  |  |  | No. of MII oocytes | No. of 2PN | No. of cleaved 2PN | No. of transferable embryo | No. of good-quality embryo | No. of MII oocytes | No. of 2PN | No. of cleaved 2PN | No. of transferable embryo | No. of good-quality embryo |  |  |
| 1 | 29 | 1.72 | 8 | 3 | 3 | 3 | 2 | 2 | 3 | 3 | 3 | 3 | 2 | 6 | normal |
| 2 | 27 | 1.74 | 11 | 6 | 6 | 6 | 4 | 4 | 5 | 5 | 5 | 5 | 3 | 6 | normal |
| 3 | 31 | 1.73 | 13 | 6 | 6 | 2 | 2 | 2 | 5 | 5 | 4 | 1 | 1 | 6 | normal |
| 4 | 32 | 6.72 | 15 | 5 | 5 | 3 | 2 | 2 | 4 | 4 | 4 | 2 | 1 | 6 | normal |
| 5 | 30 | 4.33 | 15 | 6 | 4 | 4 | 1 | 1 | 5 | 5 | 5 | 2 | 2 | 5 | normal |
| 6 | 33 | 4.06 | 10 | 4 | 4 | 3 | 2 | 2 | 5 | 5 | 2 | 2 | 0 | 5 | normal |
| 7 | 32 | 1.16 | 11 | 6 | 5 | 3 | 1 | 1 | 4 | 4 | 2 | 0 | 0 | 4 | normal |
| 8 | 30 | 1.10 | 8 | 3 | 3 | 1 | 1 | 1 | 2 | 0 | 0 | 0 | 0 | 4 | normal |
| 9 | 41 | 1.64 | 6 | 1 | 1 | 1 | 1 | 1 | 2 | 1 | 1 | 0 | 0 | 3 | normal |
| 10 | 26 | 2.95 | 13 | 6 | 6 | 6 | 4 | 4 | 6 | 6 | 6 | 5 | 4 | 2 | normal |
| 11 | 33 | 4.57 | 19 | 7 | 6 | 5 | 5 | 3 | 6 | 3 | 3 | 0 | 0 | 2 | normal |

Supplementary Table 3 Detailed embryonic outcomes of patients with live births following control embryo transfer

| Patient number | Age (y) | AMH (ng/mL) | No. of oocytes retrieved | MIT group | | | | | Control group | | | | | Child's age (As of 2025) | Intelligence quotient and health condition |
| --- | --- | --- | --- | --- | --- | --- | --- | --- | --- | --- | --- | --- | --- | --- | --- |
|  |  |  |  | No. of MII oocytes | No. of 2PN | No. of cleaved 2PN | No. of transferable embryo | No. of good-quality embryo | No. of MII oocytes | No. of 2PN | No. of cleaved 2PN | No. of transferable embryo | No. of good-quality embryo |  |  |
| 1 | 34 | 5.78 | 23 | 10 | 7 | 5 | 4 | 3 | 9 | 7 | 3 | 2 | 2 | 6 | normal |
| 2 | 30 | 3.76 | 13 | 6 | 6 | 1 | 0 | 0 | 5 | 3 | 2 | 1 | 1 | 6 | normal |
| 3 | 39 | 2.53 | 8 | 4 | 4 | 4 | 2 | 1 | 4 | 4 | 3 | 3 | 3 | 5 | normal |
| 4 | 32 | 5.43 | 9 | 3 | 2 | 0 | 0 | 0 | 3 | 3 | 2 | 1 | 1 | 4 | normal |
| 5 | 28 | 2.46 | 14 | 5 | 2 | 2 | 0 | 0 | 5 | 4 | 3 | 2 | 1 | 6 | normal |
| 6 | 35 | 2.70 | 12 | 5 | 4 | 3 | 1 | 1 | 3 | 3 | 3 | 2 | 2 | 6 | normal |
| 7 | 36 | 3.34 | 15 | 4 | 3 | 3 | 0 | 0 | 3 | 3 | 3 | 2 | 1 | 6 | normal |
| 8 | 35 | 9.37 | 17 | 6 | 4 | 1 | 1 | 0 | 6 | 4 | 3 | 1 | 0 | 5 | normal |
| 9 | 33 | 1.51 | 14 | 3 | 2 | 0 | 0 | 0 | 4 | 4 | 2 | 2 | 1 | 6 | normal |
| 10 | 31 | 2.35 | 16 | 6 | 5 | 4 | 2 | 1 | 5 | 4 | 3 | 3 | 2 | 5 | normal |
| 11 | 35 | 1.91 | 7 | 3 | 3 | 2 | 2 | 1 | 2 | 2 | 2 | 2 | 2 | 5 | normal |
| 12 | 33 | 2.66 | 14 | 6 | 1 | 1 | 0 | 0 | 7 | 6 | 4 | 3 | 2 | 5 | normal |
